# Case Report: Variations in the *ALPL* Gene in Chinese Patients With Hypophosphatasia

**DOI:** 10.3389/fgene.2021.732621

**Published:** 2021-10-12

**Authors:** Qiang Zhang, Zailong Qin, Shang Yi, Hao Wei, Xun zhao Zhou, Fei Shen

**Affiliations:** ^1^ The Maternal and Child Health Care Hospital of Guangxi Zhuang Autonomous Region, Guangxi Birth Defects Prevention and Control Institute, Nanning, China; ^2^ Laboratory of Genetic Metabolism Center, Maternal and Child Health Hospital of Guangxi Zhuang Autonomous Region, Nanning, China

**Keywords:** hypophosphatasia, whole-exome sequencing, abnormal bone ossification, TNSALP, ALPL gene

## Abstract

**Background:** Hypophosphatasia (HPP) is an autosomal genetic disorder characterized biochemically by abnormal of bone parameters and serum alkaline phosphatase (ALP) activity as well as clinically by deficiency of teeth and bone mineralization. The clinical presentation is a continuum ranging from a prenatal lethal form with no skeletal mineralization to a mild form with late adult onset presenting with non-pathognomonic symptoms. ALP deficiency is the key to the pathogenesis of abnormal metabolism and skeletal system damage in HPP patients.

**Methods:** We investigated five patients with skeletal dysplasia in the clinic. Whole-exome sequencing was performed in order to aid diagnosis of the patients.

**Results:** Eight variants in the *ALPL* gene in the five unrelated Chinese patients (PA-1: c.649_650insC and c.707A > G; PA2: c.98C > T and c.707A > G; PA3: c.407G > A and c.650delTinsCTAA; PA4: c.1247G > T (homozygous); PA5: c.406C > T and c.1178A > G; NM_000478.5) were found. These variations caused two types of HPP: perinatal HPP and Odonto HPP. All cases reported in this study were autosomal recessive. Among the variants, c.1247G > T/p.Gly416Val (PA-4); c.1178A > G/p.Asn393Ser (PA-5) and c.707A > G/p.Tyr236Cys (PA-1, PA-2) have never been reported before.

**Conclusion:** Clinical phenotypes of perinatal HPP (PA-1,PA-2,PA-3 and PA-4) include skeletal dysplasia, shorter long bones, bowing of long bones, tetraphocomelia, abnormal posturing and abnormal bone ossification. Odonto HPP (PA-5) only presents as dental abnormality with severe dental caries and decreased ALP activity. Our study extends the pool of *ALPL* variants in different populations.

## Introduction

Hypophosphatasia (HPP, OMIM: 146300, 241500, 241510) is primarily caused by *ALPL* gene variations (OMIM: 171760). So far, at least 411 *ALPL* gene variants have been reported. These variations can have obvious heterogeneity and can be inherited in an autosomal dominant (AD) or recessive (AR) manner (http://alplmutationdatabase.hypophosphatasie.com/). *ALPL* is located on chromosome 1p36.1 and consists of 12 exons distributed over 50 kb, encoding for a tissue non-specific alkaline phosphatase (TNSALP) (Xu et al., 2018). TNSALP is a homodimeric enzyme where each monomer is composed of 524 amino acids (57.2 kDa). Under physiological conditions, it functions as an ecto-phosphatase and it hydrolyzes inorganic pyrophosphate (PPi) to phosphate (Pi) to form hydroxyapatite, and this balance is essential for the bone mineralization. A loss of function variation within the gene *ALPL* will reduce the alkaline phosphatase (ALP) activity, which can lead to an accumulation of its substrates including pyridoxal-5-phosphate (PLP) and PPi. Increased PPi levels can causes impaired skeletal mineralization by blocking hydroxyapatite crystal formation, thus predisposing an individual to fractures, fracture healing complications, and bone marrow edema (BME) (Mao et al., 2019; Orimo, 2010; Weiss et al., 1988b; Zhou et al.,and 2012).

Although HPP is rare, but it is relatively common among Canadian Mennonites. The prevalence in the white-skinned populations of the United States is higher than those of black origins. The ethnic groups of the patients involved in this study were Han (PA-1, 2, 3 and 5) and Zhuang (PA-4). HPP has also been reported in Hispanics, Japanese and Chinese populations, but the prevalence of HPP in China is still unknown (Whyte, 2017; Mao et al., 2019). The severity of HPP clinical manifestations varies greatly. Patients can manifest this disease as abnormal bone mineralization, tooth or joint disease, muscle weakness, etc. A classification based on the age of diagnosis is used to define the disease: perinatal lethal (ORPHA: 247623), prenatal benign (ORPHA: 247638), infantile (ORPHA: 247651), childhood ORPHA: 247667), adult (ORPHA: 247676), and Odonto HPP (ORPHA: 247685) (Mornet, 2007; Hofmann et al., 2013; Mentrup et al. 2017). It should be noted that the clinical manifestations of various types of HPP may overlap and are not always classified strictly.

We report 5 cases of HPP patients in the Chinese population. These four patients were perinatal HPP and one patient was Odonto HPP. The five patients involved had eight variants of which 3 variants were novel.

## Methods

### Ethical Compliance

All procedures in this study were approved by the Institutional Review Boards and Ethics Committees of Maternal and Child Health Hospital of Guangxi Zhuang Autonomous Region (no.:2017-2-11). Informed consent was obtained the parents of each participant of the study.

### Study Participants

The five patients included four fetuses (PA-1, 2, 3, and 4), and one adolescent (PA-5). All of five patients showed abnormal skeletal development. Among them, four fetuses were found to be abnormal by ultrasound during pregnancy examination. Therefore, they were encouraged to have further examinations performed.

### Genetic Analysis

Peripheral blood samples from the patient and their parents or sibling were obtained for DNA extraction. In this study, whole-exome sequencing (WES) was only performed on the probands of each family (so-called proband-WES) to search for candidate disease-causing variants. The resulting putative pathogenic variants were confirmed by Sanger sequencing in both the proband and their family members’ DNA [their parents or older sister (PA-4/5)]. Genomic DNA extraction (Lab-Aid DNA kit, Zeesan Biotech Co., Ltd. China), target capturing (Human All Exon V5/V2 Kit, Agilent Technologies, CA) and library sequencing (HiSeq 2500 platform, Illumina, United States) were conducted by using standard protocols of kits and instruments. After sequencing, reads were aligned to an indexed human reference genome (GRCh37/hg19) with Burrows-Wheeler transformation 0.7.15-r1140. Duplicate reads were removed using Picard version 1.85 (http://picard.sourceforge.net) prior to further processing. Base recalibration and variant calling were performed using the Genome Analysis Toolkit version 2.3-4Lite. Finally, identified variants were saved in a variant call format. Identification of the causal variants was facilitated by TGex software (LifeMap Sciences, United States, version 3.4.1), which was used to annotate the selected SNVs and indels. “Rare deleterious” mutations were defined as those that met the following criteria: 1) they led to a stop-gain, stop-loss, non-synonymous, frameshift or splice-site mutation and 2) their alternative allele frequencies were each equal to or <0.5% in the Genome Aggregation Database (gnomAD). The gene reference sequence transcript used was NM_000478.5 (*ALPL*) and its pathogenicity was assessed based on the ACMG/AMP 2015 guidelines (Richards et al., 2015).

### Bioinformatics Analysis of Variations

The bioinformatics tools CADD (https://cadd-staging.kircherlab.bihealth.org/), SIFT (http://sift.jcvi.org/), mutation taster software (http://www.mutationtaster.org/), and MutPred2 (http://mutpred.mutdb.org/) were used to predict the effects of missense mutations on protein structure and function. Multiple sequence alignments of *ALPL* protein sequences in vertebrate species were generated by using Clustal W software (http://www.clustal.org/clustal2/). A 3D model of the *ALPL* was constructed by using the SWISS-MODEL (https://swissmodel.expasy.org/).

## Results

### Clinical Characteristics of the Patients with Hypophosphatasia

**FIGURE 1:**
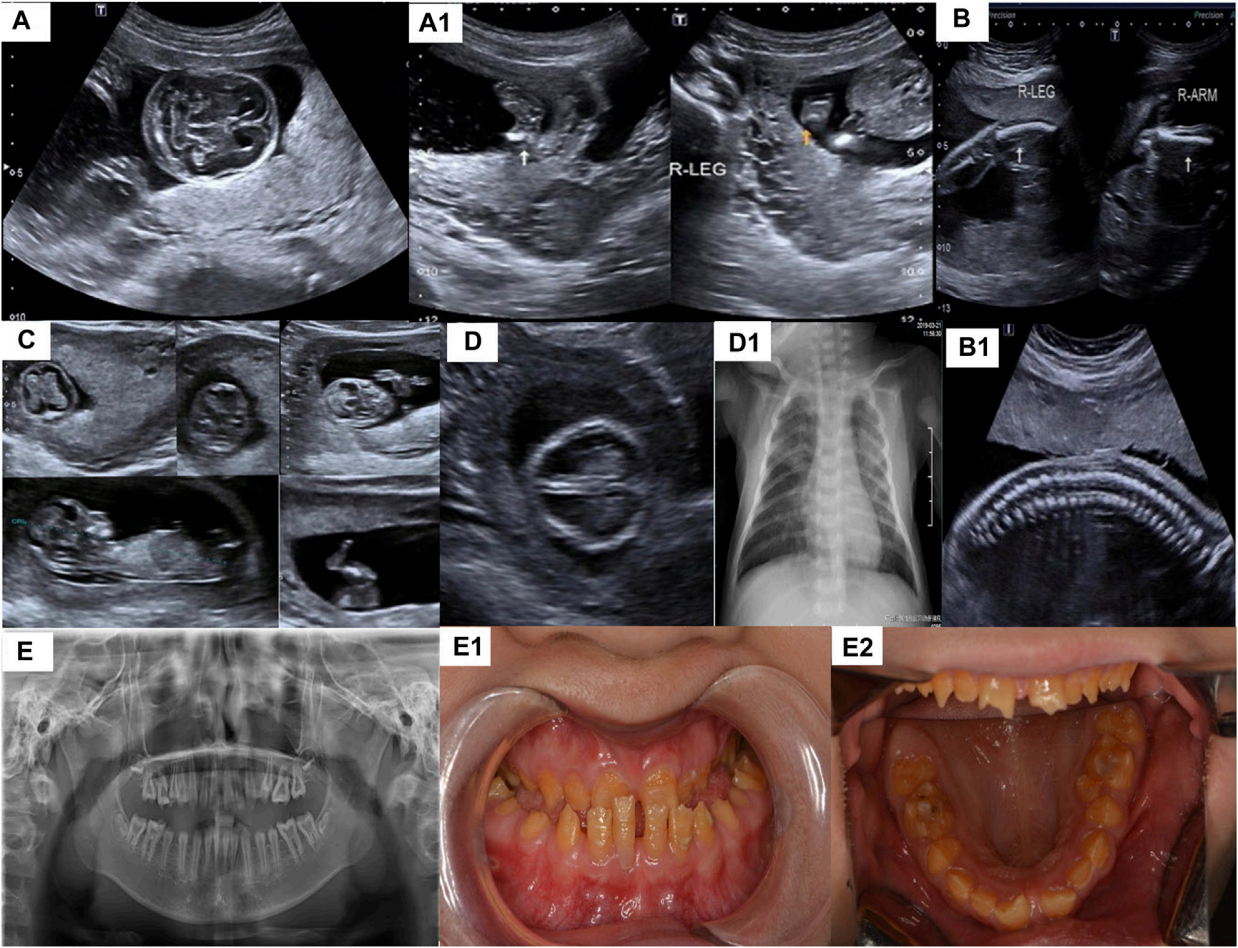
**(A,A1)** are the imaging examination results of the patient 1 (**(A)**: the echo intensity of the skull is obviously weaker than the midline of the brain, **(A1)**: Micromelia, leg posture is fixed and twisted), **(B,B1)** are the imaging examination results of patient 2 [**(B)**: short and curved femur and humerus,**(B1)**: spine had “beaded changes”] **(C)** is the imaging examination results of patient 3 (tetraphocomelia, abnormal posturing, decreased calvarial and long bones ossification, thin ribs), **(D)** is the imaging examination results of patient 4, [**(D)**: Decreased skull echogenicity. **(D1)**: pneumonia,bell-shaped thorax and some of the ribs are rachitic rosary], **(E**–**E2)** are the teeth imaging examination results and the appearance of patient 5.

PA-1 was a female fetus (17^+6^ weeks) and her mother was a 28-year-old G2P0A1 (gravida/para/abortus) woman. The result of diagnostic ultrasound exposure during the course of pregnancy showed congenital fetus malformation: tetraphocomelia, bowing of the arm and abnormal posturing. Hands and feet posture was fixed and twisted, and the echo intensity of the skull was markedly weaker than the midline of the brain. The karyotype and chromosomal microarray (Illumina Human SNP) test results of the couple were normal, but biochemical tests revealed the alkaline phosphatase concentration was low at 26 u/L (35–180 u/L). The mother had aborted a fetus in 2016 which similar symptoms but with chromosome abnormalities (for which no details were available) (Figures 1A,A1).

PA-2 was a female fetus (22 weeks) and her mother was a 22-years G1P0 woman. The B-ultrasound showed fetal malformations(short and curved femur and humerus). The length of the humerus was 22 cm, which is equivalent to 17 weeks of pregnancy and the length of the femur was 17 cm, which is equivalent to 15 weeks of gestation. There was abnormally ossified vertebrae and ossification of the skull and centrum was poor with polyhydramnios. The results of biochemical tests (blood glucose, urine and blood routine test), metabolic tests (bone metabolism) and karyotype analysis (chromosome G banding) of the pregnant woman were normal (Figures 1B,B1).

PA-3 was a female fetus (12^+5^ weeks) and her mother was a 34-years G2P0 woman. All examination results during pregnancy were normal except for the ultrasound results which showed: tetraphocomelia, abnormal posturing, decreased calvarial and long bones ossification, and thin ribs. The crown-rump length (CRL) was 6.1 cm and nuchal translucency (NT) was 0.18 mm. The patient had no family history of the disease. (Figures 1C).

**FIGURE 2:**
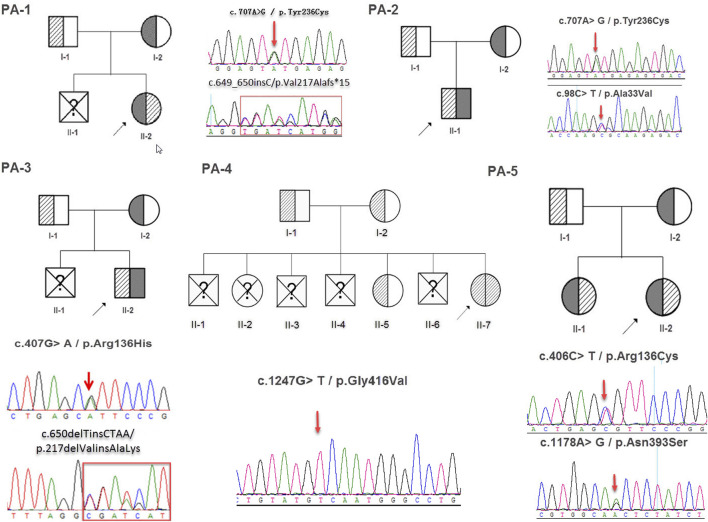
The family tree and sanger sequencing results.

PA-4 was a female fetus (28 weeks) and her mother was a 38-years G7P3 woman. The B-ultrasound showed shortened limbs and an abnormal posture as well as decreased skull echogenicity of the fetus (Figure 2, PA-4-II-7). The mother had four previous miscarriages due to fetuses with features suggestive of skeletal dysplasia. She gave birth to a boy in 2019 (Figure 2, PA-4-II-6), who weighed 3.02 kg with a height of 50 cm and with his head measuring 32 cm and a chest circumference of 32 cm. He had no history of asphyxiation after birth. The Apgar scores were 3-4-6.

A physical examination of PA-4-II-6 showed that he had slow pupillary light response, irregular respiration, dyspnea, crackles, hypokinesia, weak cry, poor suck, abdominal distention, muscular hypotonia and intracranial hypertension. Brain examination showed that the amplitude of integrated electroencephalography (AEEG) was abnormal. Head MRI results suggested that there was some hypoxic-ischemic brain damage. A cranial ultrasound scan showed sub-ependymal cysts. Chest radiographs were taken which showed pneumonia, a bell-shaped thorax and some of the ribs were rachitic rosary. Laboratory examination showed that the number of the white blood cells and platelets were increased from normal values of 4–10*10^9/L and 100–300*10^9/L to 15.4*10^9/L and 592*10^9/L, respectively. There was an increase in blood ammonia concentration (58.0 umol/L) and a decrease of the levels of alkaline phosphatase levels (24 u/L) (Figures 1D,D1). The baby boy died of respiratory failure shortly after birth and unfortunately a genetic test was not performed. From the saved test data and the genetic test results of his sister, we speculate that the boy may have had HPP.

PA-5 was a 14-year old girl, she was referred to our clinic for genetic evaluation, because of her abnormality of the dentition and premature loss of primary teeth. She has an elder sister with similar symptoms. Biochemical examination showed that the activity of ALP was significantly decreased to 9 u/L. Medical imaging examination showed carious teeth, alveolar bone loss around teeth, shortened dental roots, taurodontia and abnormality of the mandible (Figures 1E–E2).

### Genetic Analysis of Whole Exome Sequencing

Whole exome sequencing using the genomic DNA of the proband was performed in total and >18.1G clean data were generated covering 95.5% of exome target regions at least 20X. The detailed sequencing data of the variants are presented in Table 1. Based on the TGex Software (LifeMap Sciences, United States), we found variants existed in OMIM genes whose functions matched with known phenotypes. After PCR amplification, Sanger sequencing was used to identify variations. Genealogy and sequencing results are shown in Figure 2. We finally identified the cause of the disease in the five patients (PA-1: c.649_650insC and c.707A > G; PA2: c.98C > T and c.707A > G; PA3: c.407G > A and c.650delTinsCTAA; PA4: c.1247G > T (homozygous); PA5: c.406C > T and c.1178A > G; NM_000478.5), as shown in Table 2. The five patients had eight variants. Both c.1247G > T/p.Gly416Val, c.1178A > G/p.Asn393Ser and c.707A > G/p.Tyr236Cys were not presented in population and disease databases including gnomAD(https://gnomad.broadinstitute.org/), the Human Gene Mutation Database (http://www.hgmd.cf.ac.uk/ac/), the ClinVar (https://www.ncbi.nlm.nih.gov/clinvar/) and LOVD (http://www.LOVD.nl/LTBP-4). All of these were novel variants.

**TABLE 1:** Variant calling Q&R.

Patient	Variable sites	Q&R	Read depth	Read counts (ref,alt)	ALT (%)	GQ	PL	AMP score
PA-1	c.649_650insC/p.Val217Alafs*15	High	95	51, 44	46.32	99	1727,0,0	0.66
—	c.707A > G/p.Tyr236Cys	High	125	64, 61	48.80	99	1341,0,0	0.87
PA-2	c.98C > T/p.Ala33Val	High	139	86, 52	37.68	99	896,0,0	1.18
—	c.707A > G/p.Tyr236Cys	High	106	62, 42	40.38	99	782,0,0	0.90
PA-3	c.407G > A/p.Arg136His	High	113	50, 63	55.75	99	1426,0,0	0.61
—	c.650delTinsCTAA/p.217delValinsAlaLys	High	107	45, 62	57.94	99	3094,0,0	0.58
PA-4	c.1247G > T/p.Gly416Val	High	369	0, 366	100	99	10219,1094,0	2.41
PA-5	c.406C > T/p.Arg136Cys	High	153	64, 89	58.17	99	2076,0,0	1.29
—	c.1178A > G/p.Asn393Ser	High	342	180, 159	46.9	99	3360,0,0	2.87

Q&R: Classifies calls by their quality based on GQ and Coverage, the GQ mathes genotype quality and coverage matches depth. Low: Coverage <10x and GQ < 15; Med: Coverage <20x and GQ < 50; High: Coverage >=20x and GQ >=50; Alt:The percentage of reads showing the alternative allele.; GQ:Quality score based on the variant calling; PL:Genotype likelihood (HOM REF, HET, HOM ALT); AMP Score: means Amplification score (coverage/median coverage). In order to check whether the sequencing coverage of variant is abnormally high/low, we use the ratio of variants coverage to median coverage.if AMP Score >1, the depth of mutant variant is higher than median coverage; if not, the depth is less than or equal to the median coverage.Because of the preference of sequencing, some of the mutation depths may be extremely high, so the average value may not be able to reflect the intermediate level of sequencing depth. Therefore, what we choose here is the median sequencing depth instead of average depth.

**TABLE 2:** Five patients with ALPL gene variations.

Patient	Gender	Exon	Variable sites	Status	Parental validation results	Pathogenicity	Clinical form	Sensitive positions	Conservation	Sequencing primer
PA-1	Female	7	c.649_650insC/p.Val217Alafs*15^#^	Compound heterozygous	F/heterozygous	P	Perinatal	NA	NA	5’-ACC​GTC​CCA​ATA​GAC​TCG​TG-3’ 5’-CTT​CCA​GGT​GTC​AAC​GAG​GT-3’
		7	c.707A > G/p.Tyr236Cys^▲^		M/heterozygous	LP	Perinatal	Phosphorylation; Calcium site	+	5’-CTG​GAC​AAG​TAA​GGC​CCA​GA-3’ 5’-TGG​AGG​AAA​GAT​TTC​CGA​TG-3’
PA-2	Male	3	c.98C > T/p.Ala33Val^#^	Compound heterozygous	F/heterozygous	P	Perinatal	Homodimeric Interface; N-ter helix	+	5’-TTT​CTG​GAG​GAT​CTG​GAT​GG-3’ 5’-CAG​AGC​AGG​CCT​CTT​GAA​TC-3’
		7	c.707A > G/p.Tyr236Cys^▲^		M/heterozygous	LP	Perinatal	Phosphorylation; Calcium site	+	5’-CTG​GAC​AAG​TAA​GGC​CCA​GA-3’ 5’-TGG​AGG​AAA​GAT​TTC​CGA​TG-3’
PA-3	Male	5	c.407G > A/p.Arg136His^#^	Compound heterozygous	M/heterozygous	p	Perinatal	Phosphorylation	++	5’-AGT​CCC​CAT​GGT​GTG​AGT​GT-3’ 5’-GAA​AGA​CTG​AGG​CCT​GGA​CA-3’
		7	c.650delTinsCTAA/p.217delValinsAlaLys^#^		F/heterozygous	-	Perinatal	NA	NA	5’-ACC​GTC​CCA​ATA​GAC​TCG​TG-3’ 5’-CTT​CCA​GGT​GTC​AAC​GAG​GT-3’
PA-4	Male	11	c.1247G > T/p.Gly416Val^▲^	Homozygous	M/heterozygous F/heterozygous	LP	Perinatal	Crown domain	++	5’-AAG​CCA​CCA​AGG​AGC​CTA​AT-3’ 5’-TCA​TTC​TGA​GAC​CCC​TGA​CC-3’
PA-5	Female	5	c.406C > T/p.Arg136Cys^#^	Compound heterozygous	F/heterozygous	p	Odonto	Phosphorylation	++	5’-AGT​CCC​CAT​GGT​GTG​AGT​GT-3’ 5’-GAA​AGA​CTG​AGG​CCT​GGA​CA-3’
		10	c.1178A > G/p.Asn393Ser^▲^		M/heterozygous	LP	Odonto	Crown domain	+	5’-CAG​ATC​TTC​CTC​CCC​TCC​TC-3’ 5’-CTG​TCT​ACC​CGA​CCA​CCA​CT-3’

F: father; M: mother; ++: conserved residue; +: conservative changes only; NA: not applicable; ▲: novel mutations in ALPL; #: known variants in ALPL.

**FIGURE 3:**
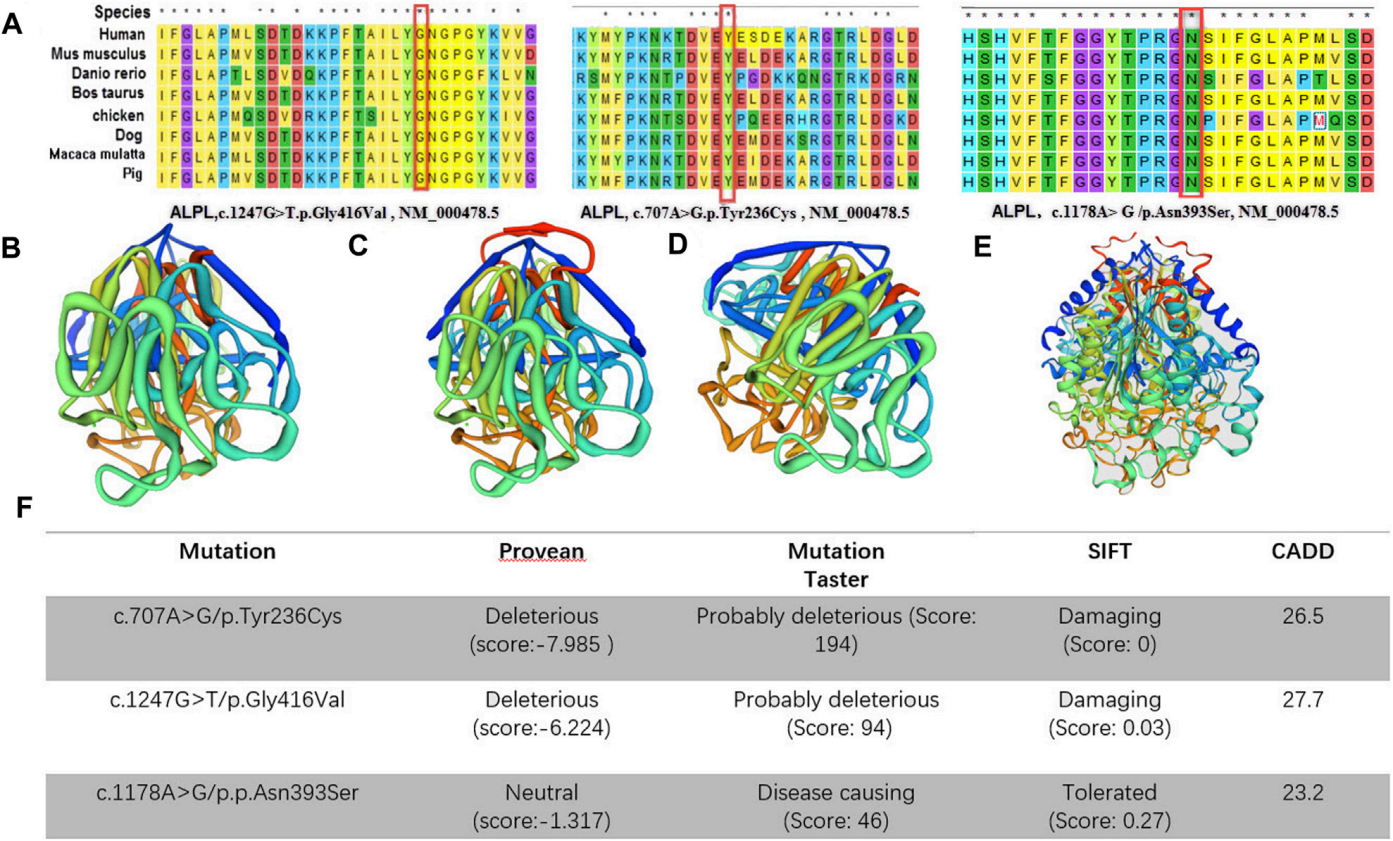
**(A)**, Conservative analysis of the variations; **(B–E)** Three-dimensional structures of alkaline phosphatase [**(B)**: wild-type, **(C)**: c.1247G > T/p.Gly416Val mutant-type, **(D)**: c.707A > G/p.Tyr236Cys mutant-type, c.1178A > G/p.Asn393Ser mutant-type]; **(F)**, in *silico* predictions. The impact of both of the *ALPL* variants was predicted using five in *silico* tools.

CLUSTAL W was used for conservative analysis of three variations (Figure 3A). Using this method, it was found that c.1247G > T/p.Gly416Val; c.1178A > G/p.Asn393Ser and c.707A > G/p.Tyr236Cys were conservative substitutions. At the same time, we predicted the impact of c.1247G > T/p.Gly416Val, c.1178A > G/p.Asn393Ser and c.707A > G/p.Tyr236Cys with four in*-silico* tools: SIFT, Provean, Mutation Taster, and CADD (Figure 3F). Predictive software suggested that c.1247G > T/p.Gly416Val and c.707A > G/p.Tyr236Cys were harmful variations, while for c.1178A > G/p.Asn393Ser, only Mutation Taster and CADD supported the possibility that this variation was harmful. The c.1247G > T/p.Gly416Val was assessed to be likely pathogenic (PM1, PM2, PM3, PP3, and PP4), c.707A > G/p.Tyr236Cys was assessed to be likely pathogenic (PM1, PM2, PM3, PP3, and PP4) and c.1178A > G/p.Asn393Ser was assessed to be likely pathogenic (PM1, PM2, PM3, PM5, and PP4) by the ACMG/AMP guidelines. In combination with the clinical manifestations of the patients and the results of the pathogenicity assessment of the variants, we finally diagnosed that these five patients were hypophosphatasia.

## Discussion

HPP is highly variable in its clinical expression and the relationship between genetic transmission and symptoms is not well understood. Even with the same mode of transmission, the symptoms and intensity of HPP can vary from person to person. Generally, the genetic model of severe HPP is autosomal recessive inheritance and mild HPP is either autosomal dominant or autosomal recessive inheritance (Etienne Mornet, 2013). The severity of HPP depends on the effect that the *ALPL* pathogenic variant has on TNSALP activity. The cases involved in this study were all autosomal recessive. The first was identified as missense variation (A162T (c.535G > A)) of *ALPL* gene by Weiss and his co-workers in 1988 (Weiss et al., 1988a). Since then, more than 400 variations have been identified. These variations are mainly distributed in exons 5 and 12. Currently, only one variation has been found within exons 1. Most of them are Missense (71.2%), other reported variations are small deletions (11%), splicing (4.9%), nonsense (4.6), small insertions (3.4%), large del/dup (2.9), Ins/Del (1.5) and regulatory (0.5%) (http://alplmutationdatabase.hypophosphatasie.com/). In this study, the missense variations accounted for 77.78% (7/8) of the variants found.

A large number of scholars have reported on *ALPL* gene variations, and they have established a database of the hypophosphatase gene variations and clinical phenotype data (http://alplmutationdatabase.hypophosphatasie.com/).This not only enriches the human disease gene pool, but also helps medical professionals to fully understand the structure and function of the gene as well as susceptibility to the disease. In this study, we report three new variants, which can enrich the HPP variation spectrum. There are five important domains in the TNSALP molecule: active site, active site valley, homodimer interface, crown domain and calcium-binding site. When variations occur in or near these areas, they may affect the activity of the protein. Silvent et al. found 469 possible sensitive positions of the 524 residues of human TNSALP, which indicates a highly constrained protein (Silvent et al., 2014). Any substitution occurring at one of these positions is predicted to lead to HPP. Depending on the results of the study, we evaluated three novel variants (c.1247G > T/p.Gly416Val, c.1178A > G/p.Asn393Ser and c.707A > G/p.Tyr236Cys). These are in the crown domain and the calcium and phosphorylation sites. Through the tertiary structure model of the protein, we know that loss of phosphorylation results in gaining a disulfide linkage results in an altered transmembrane protein at Y236. Also, loss of the catalytic site results in altered metal binding and altered ordered interface at N417. Altered transmembrane protein results in an altered ordered interface at N393 (Figures 3B–E). It was reported in the literature that amino acid changes near these novel variants (p.Tyr236Cys, p.Gly416Val and p.Asn393Ser) will cause changes in ALP activities, such as p.E235A, p.N417S and p.R391C, respectively (Fauvert et al., 2009; Sultana et al., 2013; Komaru et al.and, 2019). Therefore, the three variants were located in a mutational hot spot and/or critical and well-established functional domain (PM1). In addition, all of them were absent from controls (or at extremely low frequency if recessive) in Exome Sequencing Project, 1000 Genomes Project (PM2). Also, for recessive disorders, which was detected in the transcript with a pathogenic variant (PM3) (Table 2). Finally, multiple lines of computational evidence supported a deleterious effect on the gene or gene product (PP3) (Figure 3).

According to ACMG guidelines, the three variants can also be assessed as likely pathogenic variants. However, it has been pointed out that very low values of residual enzymatic activity were associated with the more severe clinical presentations of HPP. For example, the previously reported mutation c.98C > T/p.Ala33Val (PA-2) found in this study, which has a 12.0% residual activity of the wild type protein (Del Angel et al.and, 2020). So, when it constitutes a compound heterozygote with c.707A > G/p.Tyr236Cys (PA-2), it can cause the patient to exhibit clinical symptoms during the fetal period. Based on these circumstances, we had reasons to believe that the three novel variants are likely to be pathogenic variants of *ALPL*. However, it is important to point out that this evidence is only theoretical. In order to analyze the effects of the novel variants on mRNA processing, expression experiments using variant cDNAs from patients would be a more efficient method that could provide more direct evidence for pathogenicity assessment. Unfortunately, these types of experiments are not possible to perform at present due to the limited resources at our institution.

It is envisaged that *ALPL* genetic analysis will be an important method for HPP diagnosis, especially when clinical manifestations and medical imaging examination cannot confirm HPP during the fetal period. Some scholars even believe the screening of variations is a more reliable indicator than the detection of serum ALP activity, but attention should also be paid to the occurrence of certain new variation types (E. Mornet, 2000). Currently, inconsistencies in genotype-phenotype correlations have been observed in some patients, indicating that other genetic or environmental factors regulate the phenotypes of this disease (Mornet, 2018).

Most patients with HPP have unique genotypes, making genotype-phenotype correlations difficult to assess (Choida and Bubbear, 2019). However, site-directed mutagenesis experiments have identified alleles producing significant residual enzymatic activity as well as alleles showing a dominant negative effect. Less severe phenotypes have been correlated with alleles that allow residual enzymatic activity in recessive HPP, and with alleles exhibiting a dominant negative effect in dominant HPP (Fauvert et al., 2009).

In this study, five patients with *ALPL* gene variations were found, involving two disease types (perinatal HPP and Odonto HPP). (Table 2) By summarizing the four cases of perinatal HPP reported in this study, we have learned that perinatal HPP children can be examined using ultrasound and may show asymmetric bone abnormalities, excessive amniotic fluid, significant and severe mineralization damage, short-limb dwarfism, long bones bending, and bone density reduction. Four patients with perinatal HPP were found to be abnormal by imaging at early stages of pregnancy. It can be observed that ultrasound imaging during pregnancy can catch the disease early. Early diagnosis may allow clinicians to provide antenatal interventions earlier. This can play a huge role in the reducing birth defects (Conner et al., 2014; Rayburn et al., 2015). In addition, perinatal HPP is divided into severe type and benign types, so there is a need caution during diagnosis of the fetus. Genetic diagnosis should be combined with imaging findings, and severe type HPP should be considered when the fetus exhibits severe bone mineralization deficiency, or the presence of a microthorax. Generally, the earlier and more obvious the impairment of skeletal mineralization appears, the more serious the disease is.

In this study, Odonto HPP (PA-5) was manifested as hypoplasia of the dental root, dentinogenesis imperfecta and taurodontia, abnormality of the periodontium and irregular tooth absorption, which are similar to the symptoms of previously reported cases (Comut et al., 2001; Wang et al., 2004; Mori et al., 2016). Previous studies have also revealed that hypophosphatase can cause complete loss or severe reduction of cementum covering the surface of the tooth root (van den Bos et al., 2005; Zweifler et al., 2015). Cementum loss hinders the normal fixation of periodontal ligament fibers and causes premature loosening and loss of teeth. Patients with HPP have reduced alkaline phosphatase activity and lower ability of dental pulp cells to form mineralized nodules, which may promote dentinal dysplasia. About 3/4 of the affected children have premature deciduous teeth loss. Primary incisor loss usually occurs before the age of 4, and more serious cases this can occur as early as 18 months of age (Chapple, 1993).

The diagnosis and classification of HPP requires a combination of tests. In this study, the diagnosis of the disease was based on the patient’s clinical manifestations, laboratory tests, and identification of pathogenic variations. In future, HPP should be considered as a possibility if a patient has abnormal ultrasound imaging of the skeletal system. But for prenatal cases, ultrasound imaging provides limited help in the diagnosis of HPP. In addition to HPP, type II osteogenesis imperfecta (OI) and, flexural dysplasia, and hypophosphatemia should be considered. It is a challenge for even experienced sonographers to differentiate between these disorders. Therefore, a definitive diagnosis of this disease needs to be made with the help of genetic testing, but classification depends on the effect that the *ALPL* pathogenic variant has on TNSALP activity as well as imagological examination. Furthermore, for postpartum cases, it should be noted that decreased ALP activity can also occur in other diseases, such as hypothyroidism, celiac disease, malnutrition, anemia and so on (Nzioka Mutua et al., 2018). We should monitor ALP activity and consider HPP only when the ALP activity is persistently low. Meanwhile, in order to distinguish these disorders, the levels of phosphoethanolamine, pyridoxal phosphate, or pyrophosphate are important parameters which need to be evaluated.

At this stage, therapeutic options are mainly symptomatic treatment and prevention of potential diseases. In the literature, bone marrow stem cell transplantation can improve the clinical symptoms and imaging performance of children during their childhood, but these are unlikely to improve the perinatal HPP (Katsube et al., 2010). Enzyme replacement therapy (ERT) is considered to be the best treatment method with a clear effect so far, but these drugs have yet to be introduced in China. Unfortunately, in our study, the parents of perinatal HPP fetuses were unwilling to take the risk after birth and eventually decided to abort the pregnancies. Children with odontogenic HPP received regular dental follow-ups and treatments (such as the introduction of dentures). The diagnosis and treatment schedule of the patients were presented in Table 3.

**TABLE 3:** A timeline with diagnosis and treatment.

Patient	Date	Diagnosis process	Intervention	Follow-up
PA-1	2-May-2018	Ultrasound abnormalities in 17^+6^ weeks, laboratory testing and gene test(WES)	Labor induction (11-Jul-2018)	Pregnancy (12-May-2021)
PA-2	22-Oct-2019	Ultrasound abnormalities in 22 weeks, laboratory testing and gene test(WES)	Labor induction (30-Dec-2019)	Pregnancy preparation (6-Feb-2021)
PA-3	8-Feb-2018	Ultrasound abnormalities in 12^+5^ weeks, laboratory testing and gene test(WES)	Labor induction (16-May-2018)	Pregnancy preparation (1-Mar-2020)
PA-4	28-Jun-2020	Ultrasound abnormalities in 28 weeks, laboratory testing and gene test(WES)	Labor induction (29-Jul-2020)	No fertility plan (25-Jun-2021)
PA-5	16-Jun-2020	Abnormality of the dentition and premature loss of primary teeth, laboratory testing and gene test(WES)	Symptomatic treatment (7-Aug-2020)	Dentures (25-Jun-2021)

## Conclusion

HPP is a highly heterogeneous disease. With this type of study, clinicians can deepen their understanding of the disease. The study identifies three novel variants of the *ALPL* gene in Chinese patients, which can enrich the HPP variation spectrum and extend the phenotype spectrum of the disease in different ethnic groups and this will help improve future variation-based screening and genetic diagnosis.

## Data Availability

The datasets presented in this study can be found in online repositories. The names of the repository/repositories and accession number(s) can be found in the article/Supplementary Material.
